# A Genomic Approach to Study Anthocyanin Synthesis and Flower Pigmentation in Passionflowers

**DOI:** 10.4061/2011/371517

**Published:** 2011-05-05

**Authors:** Lilian Cristina Baldon Aizza, Marcelo Carnier Dornelas

**Affiliations:** Departamento de Biologia Vegetal. Rua Monteiro Lobato 970, Instituto de Biologia, Universidade Estadual de Campinas, Cidade Universitária Zeferino Vaz, 13083-970 Campinas, SP, Brazil

## Abstract

Most of the plant pigments ranging from red to purple colors belong to the anthocyanin group of flavonoids. The flowers of plants belonging to the genus *Passiflora* (passionflowers) show a wide range of floral adaptations to diverse pollinating agents, including variation in the pigmentation of floral parts ranging from white to red and purple colors. Exploring a database of expressed sequence tags obtained from flower buds of two divergent *Passiflora* species, we obtained assembled sequences potentially corresponding to 15 different genes of the anthocyanin biosynthesis pathway in these species. The obtained sequences code for putative enzymes are involved in the production of flavonoid precursors, as well as those involved in the formation of particular (“decorated”) anthocyanin molecules. We also obtained sequences encoding regulatory factors that control the expression of structural genes and regulate the spatial and temporal accumulation of pigments. The identification of some of the putative *Passiflora* anthocyanin biosynthesis pathway genes provides novel resources for research on secondary metabolism in passionflowers, especially on the elucidation of the processes involved in floral pigmentation, which will allow future studies on the role of pigmentation in pollinator preferences in a molecular level.

## 1. Introduction

Anthocyanins belong to a diverse group of secondary metabolites of the phenylpropanoid class, the flavonoids, which are found in different plant species. They represent some of the most important natural pigments, which are responsible for the wide range of red to purple colors present in many flowers, fruits, seeds, leaves, and stems. Besides having great economical relevance, flower and fruit pigments play an important ecological role in the animal attraction for pollination and seed dispersal, wich is a spectacular example of coevolution between plants and animals [1–3].

**Figure 1:**
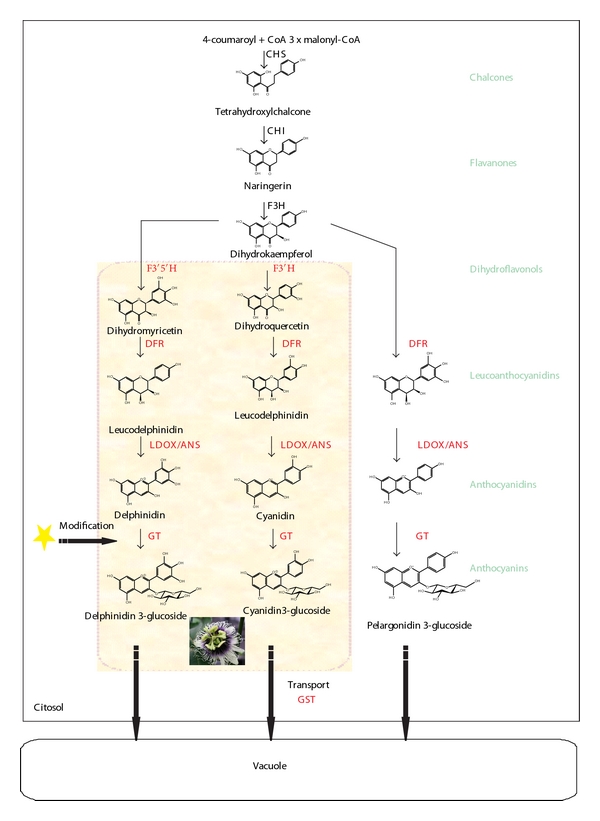
Schematic representation of the anthocyanin biosynthetic pathway (adapted from [16]). Enzymes are indicated in red, and classes of compounds are in green. Anthocyanidin is further modified with glycosyl, acyl, or methyl groups, resulting in the “decorated” anthocyanin. In this case, UF3GT is responsible for the glycosylation of anthocyanidins. The proposed anthocyanin biosynthetic pathway for *Passiflora edulis* is highlighted by the colored background. CHS: chalcone sintase; CHI: chalcone isomerase; F3H: flavanone 3-hydroxylase; F3′H: flavanone 3′-hydroxylase; F3′5′H: flavanone 3′5′-hydroxylase; DFR: dihydroflavonol 4-reductase; LDOX/ANS: leucoanthocyanidin dioxygenase/anthocyanidin synthase; GT: glucosyltransferase; GST: glutathione S-transferase.

The biosynthetic pathway of anthocyanins has been well characterized biochemically and genetically in species with different floral morphology, pigmentation pattern, and pollination syndromes such as *Petunia hybrida* [4, 5], *Matthiola* [6], *Dianthus *[7], *Eustoma *[8], *Gerbera *[9], *Zea mays* [10, 11], *Antirrhinum majus* [12], and *Ipomoea *[13, 14]. A representation of a general anthocyanin biosynthetic pathway is shown in Figure 1.

 Briefly, the pathway is initiated with chalcone synthase (CHS) catalyzing the stepwise condensation of three molecules of acetate residues from malonlyl-CoA with one molecule of 4-coumaroyl-CoA to form the basic structure of flavonoids (tetrahydroxychalcone), which is rapidly isomerized to the colorless naringenin by chalcone isomerase (CHI). Naringenin is then converted to dihydroflavonol by flavanone 3-hydroxylase (F3H). Dihydroflavonol 4-reductase (DFR), which is a specific enzyme for the anthocyanin synthesis, catalyses the production of leucoanthocyanidins from dihydroflavonols, which can be hydroxylated on the 3′ or 5′ position of the B-ring by flavonoid 3′-hydroxylase (F3′H) to produce dihydroquercetin or by flavonoid 3′5′-hydroxylase (F3′5′H) to form dihydromyricetin. Subsequently, leucoanthocyanidin oxidase/anthocyanidin synthase (LDOX/ANS) is responsible for the formation of the anthocyanidins from the colorless leucoanthocyanidins. GT enzymes (O-glucosyltransferases) represent the final step in anthocyanin biosynthesis: anthocyanidins are converted in differentially “decorated” anthocyanin molecules [15, 16]. Biochemical approaches have demonstrated that all anthocyanin pigments are derived from one of three aglycones: pelargonidin, cyaniding, and delphinidin. The main determinants of the apparent color of these pigments are the hydroxylation and methylation patterns, as well as the number and type of sugars on the beta ring of the flavonoid molecule [1, 3, 17–19].

Figure 1 depicts a generalized anthocyanin biosynthesis pathway. At least, two groups of genes are required for anthocyanin biosynthesis: the first group is represented by the structural genes encoding enzymes for the production of the flavonoid precursors, as well as those involved in the formation of particular (“decorated”) anthocyanin molecules. The second group includes the genes encoding regulatory factors that control the expression of structural genes which are mainly orenestrated by complexes formed by MYB and basic helix-loop-helix (bHLH) transcription factors that include WDR (WD40 repeats) proteins [2, 4, 15, 16, 20–23].

There are about 600 *Passiflora* species widely distributed in tropical and subtropical regions. Some *Passiflora* species have economical importance due to the production of fruits (passionfruit) or use as ornamentals. Nevertheless, a large number of* Passiflora* species are rare and/or endangered, as the environment of their diversity center has been increasingly degraded by human activities [24]. An enormous floral diversity is observed among *Passiflora* species, including variation in color, size, morphology, and fusion of floral organs. These and other floral characteristics, including evolutionary innovations such as the presence of coronal filaments and an androgynophore, are indicative of the wide range of pollination syndromes found in the genus [24]. Wide passionflowers may be pollinated by insects (bees and wasps), hummingbirds, and bats [24]. The most striking feature of floral variation among passionflowers is the wide range of pigmentation patterns of the corona filaments. Most of the floral pigments in *Passiflora* are different types of anthocyanin molecules [25, 26]. Among all* Passiflora* species, *P. edulis *Deg and *P. suberosa *L. are of particular interest, because they are model *Passiflora* species for which expressed sequences tags (ESTs) were produced within the frame of the “PASSIOMA” Project [27]. *P. edulis* Deg flowers are pollinated by large bees of genus *Xylocopa*. These flowers are about 8–12 cm wide, and their coronas contain multiple series of purplish filaments with white tips. The flowers of *P. suberosa* L. are small (2-3 cm wide) and show two morphologically distinct series of corona filaments: the outer series is greenish, and the inner series is formed by smaller purple filaments. The flowers of *P. suberosa* are pollinated by wasps [28].

We are particularly interested in the characterization of genes involved in the anthocyanin biosynthetic pathway of these two *Passiflora *species. With this aim, we searched for putative *Passiflora* genes responsible for flower pigmentation, using the key proteins known to be involved in the different enzymatic steps of anthocyanin biosynthesis as baits to search for expressed sequences tags (ESTs) in the PASSIOMA database.

## 2. Material and Methods

### 2.1. Searching Passiflora ESTs Homologous to Anthocyanin Biosynthetic Genes

The clustered expressed sequence tags (ESTs) from the PASSIOMA Project database [27] were used as a primary source of data for our analyses. These sequences were assembled from ESTs obtained from the sequencing of several *P. edulis* or *P. suberosa* cDNA libraries, made from floral buds at different developmental stages (see [27] for details on library construction, sequencing, and database structure). Nucleotide sequences and their respective deduced amino acid sequences from genes known to be involved in anthocyanin biosynthesis (see Figure 1) were obtained from the National Center for Biotechnology Information (NCBI; http://www.ncbi.nlm.nih.gov/). Searches for putative homolog sequences in the PASSIOMA database were conducted using the tBLASTN module that compares the consensus amino acid sequence with a translated nucleotide sequences database [29]. We generally used *Arabidopsis thaliana *or *Petunia hybrida* as query consensus sequences as the anthocyanin biosynthesis pathways in these model species are more thoroughly studied at the molecular level [30–32]. All sequences in the PASSIOMA database that exhibited a significant alignment (*e*-value lower than 10–5) with the query were retrieved from the PASSIOMA database.

The clusterization of all reads identified using a given query sequence was performed using the CAP3 algorithm [33] from the BioEdit software [34]. The novel cluster consensus sequences obtained were reinspected for the occurrence of conserved motives using InterProScan [35] and were compared to NCBI databases using BLAST [29]. Sequences that did not show the main motives present in the query sequence were discarded. Validated sequences were then included in phylogenetic analyses.

### 2.2. Comparison of the Amino Acid Sequences and Phylogenetic Analysis

All amino acid sequences were aligned by CLUSTALX software using default parameters [36]. The obtained alignments were eventually corrected by hand and imported into the molecular evolutionary genetics analysis (MEGA) software [37]. Phylogenetic trees were obtained using parsimony and/or genetic distance calculations (in the later case using pairwise deletion option and with the Poisson correction model). Neighbor-joining [38] and Bootstrap (with 10,000 replicates) trees were also constructed.

## 3. Results

The cDNA libraries of the PASSIOMA Project were obtained from mRNA extracted from floral buds at different developmental stages, and it is expected that all EST sequences correspond to genes expressed during *Passiflora* flower development [27]. This sequence search detected a total of 75 *Passiflora* EST sequences, 34 of them corresponding to *P. edulis* sequences and 41 of them corresponding to sequences derived from *P. suberosa* libraries. When submitted to the CAP3 algorithm and detailed comparison of their deduced amino acid sequences, the number of valid clusters was reduced to 15, potentially corresponding to 15 different genes. When the validated amino acid sequences obtained from the PASSIOMA database were compared to other plant protein sequences in the public databases, the first BLAST hits generally corresponded to *Populus* and *Ricinus* sequences. This was expected, as *Passiflora* and these genera belong to the same order (Malpighiales) and are considered to be closely related [39].

We obtained assembled EST sequences corresponding to genes of the following genes families: CHS, DFR, GT, GST, MYB, and WD40 (see Table 1). Therefore, we used 15 *Passiflora* assembled sequences from the PASSIOMA database and a selected set of genes from divergent plant species from the public databases to explore their evolutionary relationships. The obtained sequence comparison alignments allowed the construction of phylogenetic trees for each of these families of genes involved in the different enzymatic steps of the anthocyanin pathway.

The similarities among all genes identified in this study and those reported from other plant species were assembled in Table 1 and ranged from 70% (PACEPE3030G03.g; representing a putative member of the GST, glutathione S-transferase superfamily) to 96% (PACEPE3007G07.g; potentially encoding a WD40 protein).

Some of these gene sequences showed significant similarity to elements required for early or late steps of the pathway; others putatively encode regulatory proteins involved in the control of the spatial and temporal patterns of pigmentation, while others are responsible for intracellular transport of the anthocyanin molecules. The role of each of these genes in the anthocyanin biosynthesis and the probable implications for the understanding of the *Passiflora* flower pigmentation are presented in the Discussion.

### 3.1. Identification and Phylogenetic Analysis of Passionflower Genes Potentially Involved in Anthocyanin Biosynthesis and Transport

#### 3.1.1. Chalcone Synthases (CHSs)

**Figure 2:**
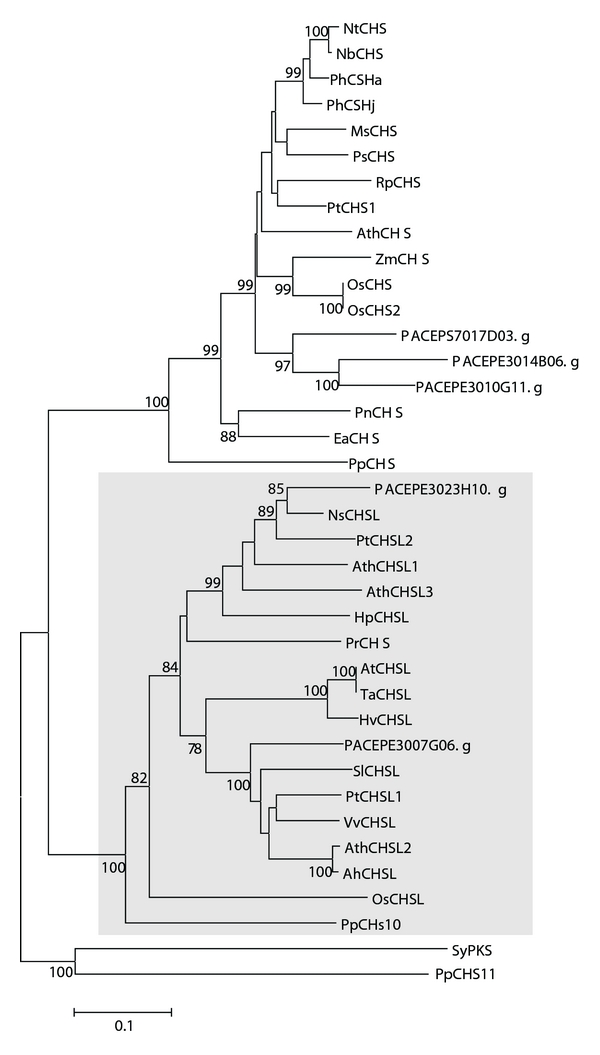
A Neighbor-joining phylogenetic tree of chalcone synthase (CHS) amino acids sequences. The cluster containing all anther-specific CHS-like enzymes is highlighted. Bootstrap values from 1,000 replicates were used to assess the robustness of the trees. Only bootstrap values above 75% are indicated at the nodes. Accession numbers for genes from other species are given in Supplementary data.

We have found 5 *Passiflora* assembled sequences (5 putative genes) encoding enzymes of the CHS family: PACEPE3010G11.g, PACEPE3014B06.g, PACEPE3007G06.g, PACEPE3023H10.g and PACEPS7017D03.g. These sequences are expected to encode proteins with 231, 158, 254, 237, and 222 amino acids, respectively. The deduced CHS proteins showed more than 80% similarity to CHSs of other plant species (Table 1). To determine the phylogenetic relationship of different CHSs, we aligned protein sequences from a diverse range of plant species (moss, ferns, gymnosperms and angiosperms), cyanobacterium (*Synechococcus* sp.) and *Passiflora* representatives of the CHS superfamily (Figure 2). The phylogenetic tree was resolved in three clades. These three clades were highly supported with 100% bootstrap values. The *Passiflora* proteins were consistently positioned into different clades. One of these monophyletic clades (highlighted in Figure 2) contains all the anther-specific CHS-like genes (ASCLs; [40, 41]). The remaining sequences, including three *Passiflora* members, were clustered in the other sister clade together with all CHS genes from seed plants.

#### 3.1.2. Dihydroflavonol 4-Reductases (DFR)

**Figure 3:**
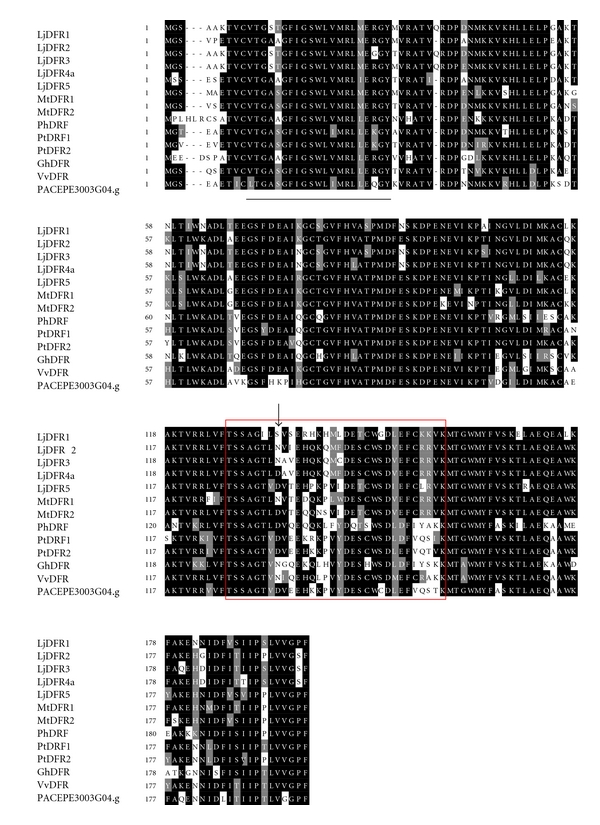
Multiple sequence alignment of the *Passiflora *sequence with some plant DFR sequences. The identical and similar residues are highlighted on a black and gray background, respectively. NADP-binding domain is underlined. Boxed amino acids have been considered to control the substrate specificity of DFR enzyme [40], and the amino acid residue (indicated by an arrowhead) is especially important for this specificity [41]. The alignment was performed using CLUSTALX and BOXSHADE program.

A single *Passiflora* cDNA sequence of 850 bp encoding a predicted protein of 204 amino acids showed significant *e*-value (1*e* ^−95^) and 94% similarity to a *Populus* DFR sequence (Table 1).  Figure 3 shows an alignment of the deduced amino acid sequence of the *Passiflora *DFR with some other plant sequences containing an NADP-binding domain, considered the region of substrate preference of DFR enzymes [42, 43]. Additionally, the *Passiflora* DFR showed an aspartic acid residue at position 134, as it is observed for the *Petunia *and* Populus *proteins, whereas *Gerbera* and some *Lotus* DFR show an asparagine residue at the same position (Figure 3). We adopted the terminology suggested by Shimada and coworkers [44] to designate the conserved motifs present in the DFR sequence.

**Figure 4:**
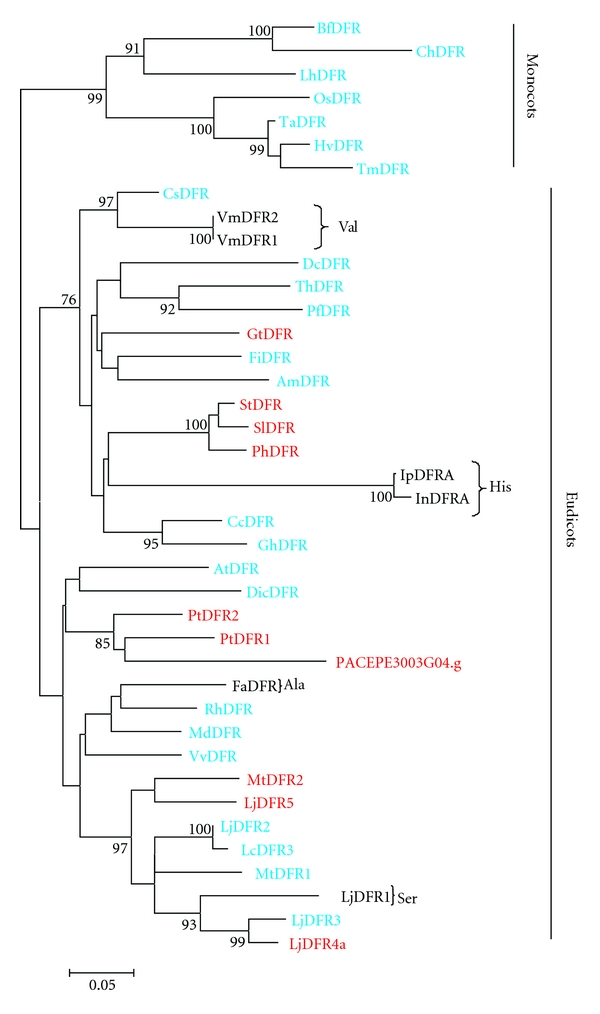
A Neighbor-joining phylogenetic tree of dihydroflavonol 4-reductase (DFR) amino acids sequences. Bootstrap values from 1,000 replicates were used to assess the robustness of the trees. Only bootstrap values above 75% are indicated at the nodes. Asn-type DFRs, Asp-type DFRs, and DFRs of neither Asn nor Asp-type are indicates in blue, red, and black, respectively [42]. Accession numbers for genes from other species are given in Supplementary data which are available online at doi:10.4061/2011/37157.

A neighbor-joining tree was constructed based on the alignment DFR sequences shown in Figure 3. The monocots and eudicots DFRs were positioned separately. While monocot DFR genes formed one clade, the eudicot DFR sequences diverged into two clades. Clearly, Asn-type DFRs are found in a larger number of species. On the other hand, Asp-type DFRs are restricted to some species, including *Passiflora* and *Populus* (Figure 4).

#### 3.1.3. Glucosyltransferases (GT)

**Figure 5:**
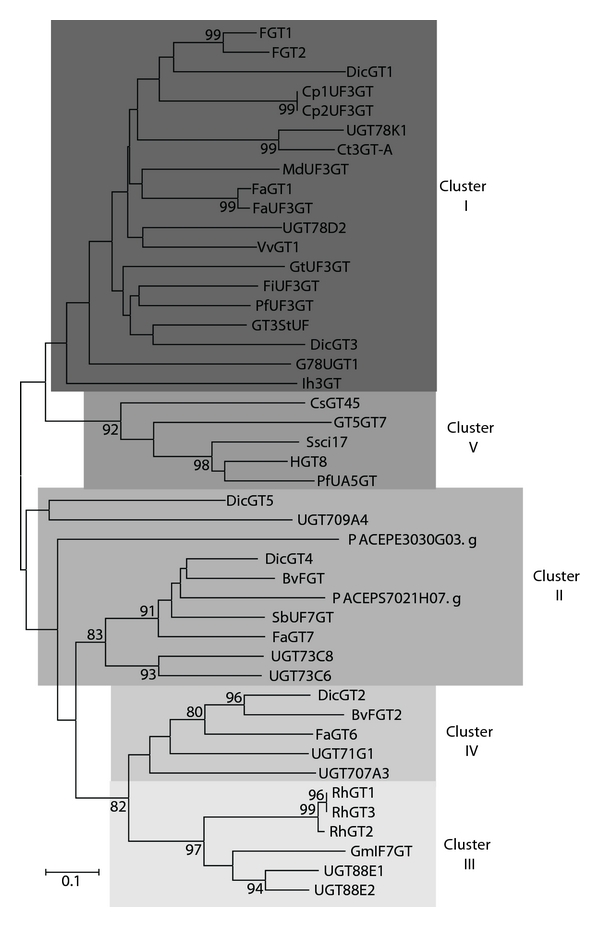
A Neighbor-joining phylogenetic tree of glucosyltransferase (GT) amino acids sequences. Bootstrap values from 1,000 replicates were used to assess the robustness of the trees. Only bootstrap values above 75% are indicated at the nodes. Accession numbers for genes from other species are given in Supplementary data.

We identified two *Passiflora* EST clones, PACEPE3030G03.g and PACEPS7021H02.g, encoding proteins with sequence similarity to *Ricinus communis* glucosyltransferases (Table 1). The first cDNA sequence contained an ORF specifying a 124 amino acid protein, and the second cDNA encoded a protein of 200 amino acid residues. These putative* Passiflora* GT proteins were compared with those GT enzymes described by Kovinick and colleagues [45] and retrieved from the NCBI database. The obtained phylogenetic tree resulted in five clades, according to their *in vitro* substrate specificities [45]. Phylogenetic analysis revealed that the *Passiflora* sequences were positioned within the Cluster II proteins (Figure 5).

#### 3.1.4. Glutathione S-Transferases (GSTs)

**Figure 6:**
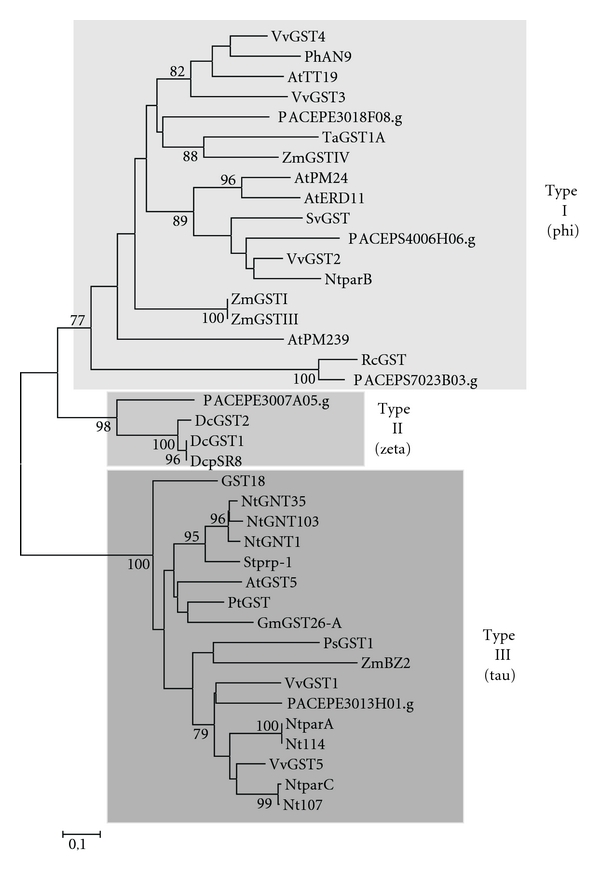
A Neighbor-Joining phylogenetic tree of glutathione S-transferase (GST) amino acids sequences with three types representing phi, tau, and zeta classes. Phi and tau are plant-specific GSTs. Bootstrap values from 1,000 replicates were used to assess the robustness of the trees. Only bootstrap values above 75% are indicated at the nodes. Accession numbers for genes from other species are given in Supplementary data.

We have identified five *Passiflora* sequences representing putative members of the GST family. Each member was represented by a single EST sequence. Comparison of these deduced GST protein sequences with those in the GenBank database revealed homology with multifunctional GSTs from *Populus*, *Ricinus*, and *Glycine* spp (see Table 1). Phylogenetic relationships among the putative *Passiflora* GSTs and family members of other plant species were established (Figure 6). Based on sequence similarity, the five *Passiflora* putative GSTs were grouped into three clades. PACEPE3018F08.g, PACEPS4006H06.g, and PACEPS7023B03.g are type I GSTs, PACEPE3007A05.g is a type II GST, and PACEPE3013H01.g is a type III GST [46].

We could not find any putative homologs to chalcone isomerases (CHI), flavanone 3-hydroxylases (F3H), and anthocyanidin synthases (ANS; see Figure 1) in the PASSIOMA database. Three EST sequences were identified corresponding to a putative flavonoid 3-O-hydroxylase (F3′H) gene, and one sequence was found that showed significant homology to genes encoding flavonoid 3-5-O-hydroxylases (F3′5′H; data not show). As these sequences were incomplete at their 5′ end, they were not considered in our analyses.

### 3.2. Identification and Phylogenetic Analysis of Passionflower Genes Potentially Involved in Spatially and Temporally Patterning Anthocyanin Deposition

Based on the searches in the PASSIOMA database, we identified one potential homolog for an MYB transcription factor of the R2R3 class. The *P. suberosa* cDNA clone PACEPS7022E07.g encodes a protein of 132 amino acids showing 91% similarity to the *Ricinus communis* R2R3 MYB. On the other hand, PACEPE3007G07.g is a putative *P. edulis* WD40 gene of 886 bp encoding 291 amino acid residues showing 96% similarity to an *R. communis*, WD40 (Table 1).

**Figure 7:**
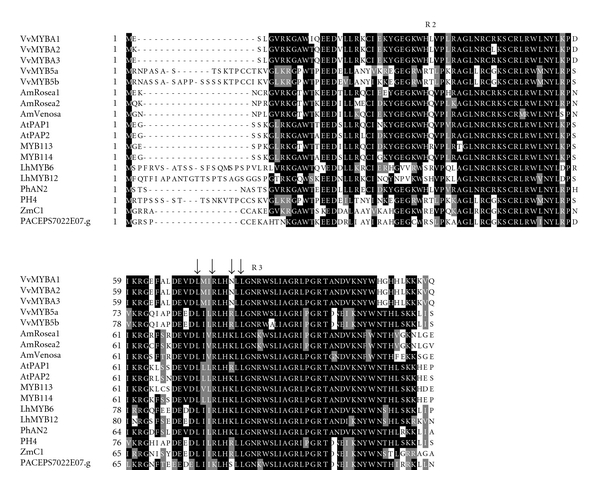
Multiple sequence alignment of the R2R3 MYB domains involved in anthocyanin production including the deduced amino acid sequence of *Passiflora suberosa*. R2R3 repeats refer to two imperfect repeats of the MYB domain. The identical and similar residues are highlighted on a black and gray background, respectively. Red box shows the R/B like bHLH interacting motif in the R3 repeat [45], and arrows indicate four specific residues of maize C1 required for interaction with a bHLH cofactor R [46]. Blue box shows a conserved motif in the R2R3 repeats for eudicots MYB related to the anthocyanin pigments [47]. The alignment was performed using CLUSTALX and BOXSHADE program.

Figure 7 shows an alignment of the deduced PACEPS7022E07.g protein sequence with 17 other plant anthocyanin-related R2R3-MYB, indicating the presence of a conserved DNA-binding domain, designated as the R2R3 domain. All sequences analyzed also contained a second conserved amino acid motif in the R3 repeat (red box), important for the interaction between MYB and bHLH proteins in *Arabidopsis* [48]. The four specific residues required for this interaction in maize [49] are also indicated by the arrows in Figure 7. The third conserved motif appears to be ANDV (blue box) in the R3 repeat of all eudicot R2R3-MYB proteins related to anthocyanin biosynthesis.

**Figure 8:**
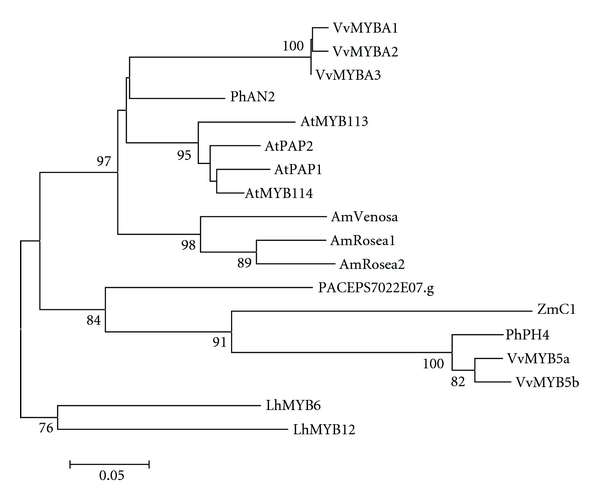
A Neighbor-joining phylogenetic tree of plant R2R3 MYB sequences. Bootstrap values from 1,000 replicates were used to assess the robustness of the trees. Only bootstrap values above 75% are indicated at the nodes. Accession numbers for genes from other species are given in Supplementary data.

A phylogenetic tree of selected plant R2R3-MYB transcription factors, including PACEPS7022E07.g, was constructed using the alignment of the conserved R2R3 repeats (Figure 8). The *Passiflora* sequence was placed within the clade including ZMC1 (*Zea mays*), PhPH4 (*Petunia hybrida*), VvMYB5a, and VvMYB5b (*Vitis vinifera*), which are known to be involved in the regulation of the anthocyanin pathway in these species [49–51].

**Figure 9:**
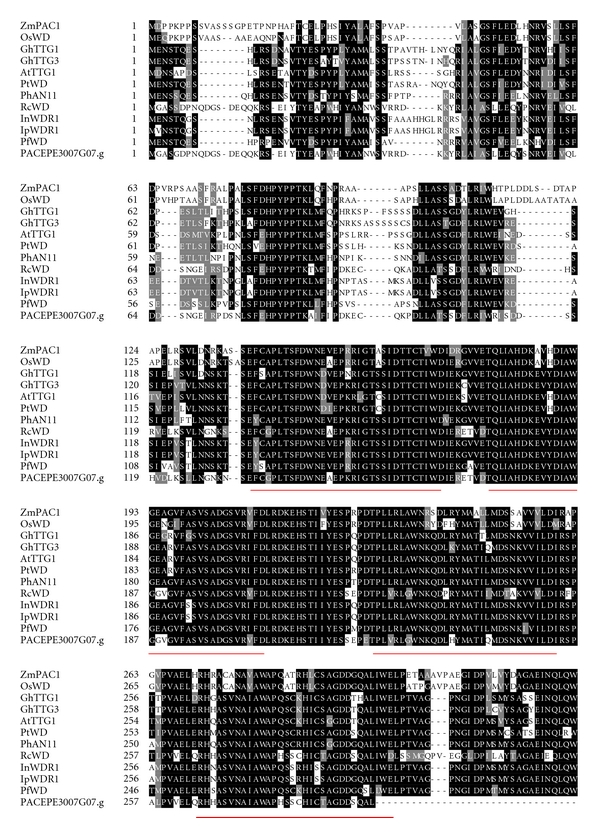
Multiple sequence alignment of the WD40 proteins involved in anthocyanin production, including the deduced amino acid sequence of the *Passiflora edulis *WD40. The identical and similar residues are highlighted on a black and gray background, respectively. Four conserved WD repeat domain are underlined in red. The alignment was performed using CLUSTALX and BOXSHADE program.

**Figure 10:**
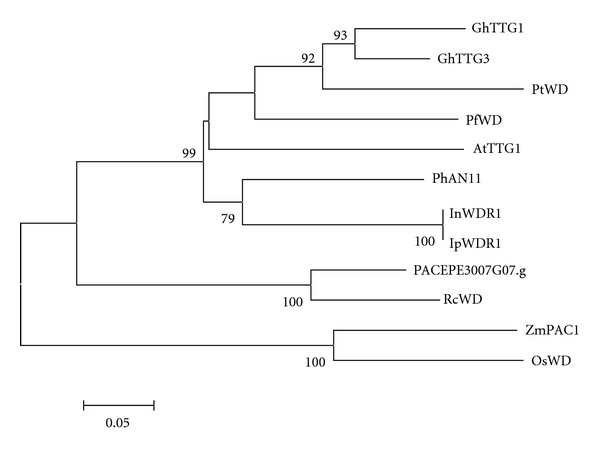
A Neighbor-joining phylogenetic tree of plant WD 40 proteins. Bootstrap values from 1,000 replicates were used to assess the robustness of the trees. Only bootstrap values above 75% are indicated at the nodes. Accession numbers for genes from other species are given in Supplementary data.

Sequence comparison of selected plant WD40 proteins with the sequence obtained from *P. edulis* indicated that the four WD repeats are highly conserved among all species analyzed (Figure 9). Phylogenetic analysis of these amino acid sequences confirmed that *P. edulis* WD40 grouped together with *Ricinus communis* WD40 and found to be more related to other dicot proteins (Figure 10).

No putative homologs to bHLH transcription factors were found in the PASSIOMA database.

## 4. Discussion

Flavonoid pathway results in the production of a range of flavonoid compounds, including anthocyanins (Figure 1). CHS is the first enzyme in the phenylpropanoid pathway and is encoded by members of a plant-specific multigene family of polyketide synthases. Nevertheless, genes belonging to the CHS family have been recently described to occur in some microorganisms (*Azotobacter vinelandii*; [52] and *Neurospora crassa*; [53]) and, therefore, indicate CHS functions might have evolved previous to the divergence of land plants. Thus, the biological functions of some of the CHS superfamily members are clearly important to plant adaptation. CHS proteins are collectively linked to the biosynthesis of different plant products with diverse functions such as UV protection, defense against pathogens, pigment biosynthesis, and pollen fertility [54, 55].

Sequence analysis indicated that two *Passiflora* CHS deduced proteins belong to a small distinct group of chalcone synthases that includes angiosperm and gymnosperms homologs to anther-specific chalcone synthase-like genes (ASCLs; highlighted in Figure 2). Furthermore, all ASCLs form a monophyletic clade. Recently, ASCLs transcripts were detected within the tapetum cells during microspore stage in wheat [56]. These genes apparently have important roles in anther development and in pollen fertility [40, 41, 56]. 

The remaining three *Passiflora* CHSs were clustered together in a sister clade containing all seed plant CHS genes. Their products are considered key in the biosynthesis of flavonoids. These include *CHSA* and *CHSJ* genes, known to be expressed in floral tissues, and involved in floral pigmentation in petunia [30, 31, 57]. Moreover, two nonchalcone genes, divergent from the typical CHSs, formed a separate clade. The *SyPKS* gene from cyanobacterium encodes an enzyme of the thiolase superfamily [58], whereas the function of the *PpCHS11* gene (from *Physcomitrella patens*) may resemble more the most recent common ancestor of all plant CHSs than do other members of the plant CHS superfamily [55].

We do not have identified putative genes encoding CHI enzymes. Besides the general limitations and drawbacks of the EST-based approach, another possible explanation may be because the rapid isomerization of chalcone to form narigen and the fact that even in the absence of a functional CHI enzyme, chalcone can spontaneously isomerize to form naringenin [15].

DFR is an enzyme catalysing the reduction of three dihydroflavonols: dihydromyricetin (DHM), dihydroquercetin (DHQ), and dihydrokaempferol (DHK) into colorless leucoanthocyanidins. These are further converted to delphinidin, cyaniding, and pelargonidin (Figure 1). The synthesis of three different anthocyanidins is mainly determined by the enzymes activities of two hydroxylases: F3′OH and F3′5′OH. The first converts DHK to DHQ and F3′5′OH converts DHK to DHM [15].

In some plant species, DFR displays distinct substrate specificity in according to the hydroxylation pattern of anthocyanin molecule [30]. A hypothesis to determine substrate specificity was proposed based on the amino acid sequence alignment of *Petunia* DFR with others plants. The alignment indicated a variable region that controls substrate recognition. Naturally, *Petunia hybrida* does not produce orange flowers, because the DFR enzyme cannot use dihydrokaempferol as substrate to produce pelargonidin, due to an aspartic acid residue at the 134th position [30, 42], as it was also observed for *Passiflora* (Figure 3), thus converting dihydroquercetin to leucocyanidin and, more efficiently, the reduction of dihydromyricetin to leucodelphinidin [30, 59]. On the other hand, some *Gerbera* genotypes have an asparagine residue at this same position and can utilize three dihydroflavonols as substrates of DFR, consequently producing orange to red colored flowers [9, 30]. Thus, the flower color is partly determined by alteration of a single amino acid that changes the substrate specificity of the DFR enzyme.

Almost all anthocyanidins undergo several modifications, which vary across species and involve enzymes of the glucosyltransferase, methyltransferase, and acyltransferase families. The most common is glycosylation of the 3-position of anthocyanidins (represented in Figure 1) to produce stable anthocyanin molecules [15, 30, 31, 60]. UDP-glucose:flavonoid 3-O-glucosyltransferase (3GT) belongs to a large multigene glucosyltransferases (GTs) family, representing the final step in anthocyanin biosynthesis.

In this work, we adopted the classification of the GTs into clusters according to Kovinic and colleagues [45]. Cluster I groups includes 3GTs enzymes. Cluster II includes GTs with multiples substrates preferences, generally for chalcones, flavones and flavonols but not anthocyanidins. Enzymes from Cluster III have isoflavone 7-O and anthocyanidin 3,5-O-GT activities. Cluster IV glycosylates flavonol and isoflavonol substrates and Cluster V have anthocyanin 5-O and/or flavonol 7-O-UGT enzymes [45]. Our results indicated that the obtained *Passiflora* glucosyltransferase gene sequences were grouped in Cluster II, together with other family members that show a high catalytic specificity for more than one class of flavonoid substrates (Figure 5). DicGT5 (from *Dianthus caryophyllus*) glycosylates a chalcononaringenin 2′-O-glucosyltransferase [61], whereas the *Beta vulgaris* GT has a favonoid-7, 4′-O-betanidin-5-O-glucosyltransferase activity [62]. Both GTs have non-anthocyanidin substrate specificity. Despite these results, obviously neither GT substrate specificity, nor *in vivo* function of the *Passiflora* GTs can be predicted solely based on amino acid sequence similarities and must be experimentally determined.

Anthocyanin biosynthesis has been demonstrated to occur predominantly in the cytosol, but these pigments are exclusively accumulated in the vacuole of epidermal cells [20]. Transport of pigments to the vacuoles requires a glutathione S-transferase and a specific carrier protein localized in the vacuolar membrane. GSTs are multifunctional proteins encoded by a large familiar present in all cellular organisms. Plants GSTs are classified on the basis of sequence identity into four classes: phi, tau, theta, and zeta [46]. The two small zeta and theta classes include GSTs from animals and plants, while the phi and tau classes are plant-specific. Several studies have confirmed the involvement of GSTs in the vacuolar transport of anthocyanins. PhAN2 (from *Petunia*), ZmBZ2 (from maize), and AtTT19 (from *Arabidopsis*) are GST proteins involved in anthocyanin transport [30–32, 63–65].

To characterize their phylogenetic relationships, the deduced amino acid sequences from the *Passiflora* putative GSTs were compared with other plant GST sequences, including the ones mentioned above. Figure 6 shows that the *Passiflora* GSTs are included into three different clades: three sequences were positioned in the same clade of PhAN9 and AtTT19 (phi class), whereas one sequence was grouped together with ZmBZ2 (tau class; [66]). Although of these known proteins belong to distinct GST clades, they perform similar functions [63–65]. 

Interestingly, PACEPE3007A05.g was clustered with carnation (*Dianthus caryophyllus*) GST type II (zeta class) which is associated to petal senescence in response to ethylene [67, 68]. 

At the moment, we can classify the *Passiflora* GSTs into type I (phi), type II (zeta), and type III (tau). At least, four of them might be involved in the anthocyanin pathway and PACEPE3007A05.g might be related to other biological processes related to flower development such as those observed for the carnation GST.

In all analyzed species, the spatial and temporal expression of the structural genes of the anthocyanin biosynthetic pathway is controlled by regulatory genes, which interfere with the intensity and pattern of anthocyanin biosynthesis [15]. MYBs, basic helix-loop-helix (bHLH) transcription factors and WD40 proteins form a transcriptional complex for the activation of the structural genes [4, 12, 20, 47, 69, 70]. MYBs and bHLHs proteins are coded by large multigene families, and those associated with anthocyanin biosynthesis are characterized by a conserved DNA-binding domain consisting of two imperfect repeats (named R2R3), and a specific bHLH domain, respectively. These two gene families have been extensively studied in model plants such as Arabidopsis and maize [48, 49, 71]. 

A multiple sequence alignment of the R2R3 domains of selected MYB proteins known to be involved in anthocyanin biosynthesis regulation, and the deduced amino acid sequence of PACEPS7022E07.g confirmed the presence of the conserved R2R3-MYB domain in this *P. suberosa* sequence (Figure 7) as well as that of a second conserved domain in the R3 repeat (red box, Figure 7), which is known to be necessary for the interaction between MYB and bHLH transcription factors [48, 49]. Additionally, a third motif in the R3 repeat (ANDV, blue box in Figure 7) represents a conserved motif shared among all eudicot MYBs involved in the anthocyanin biosynthesis [72]. 

The phylogenetic tree obtained using the alignment shown in Figure 7 is presented in Figure 8 and indicates LhMYB6 and LhMYB12 clustered outside the eudicot clade. These two genes regulate anthocyanin biosynthesis in the flowers of lily (*Lilium* hybrid), a monocot [73]. One clade is formed exclusively by eudicot anthocyanin regulators (PhAn2, AtPAP1, AtPAP2, AmROSEA1, and AmROSEA2; [12, 71, 74–77]. Curiously, one regulator of the anthocyanin in maize (a monocot), ZmC1 was positioned in the same clade of other dicot members such as PhPH4 (from *Petunia*), VvMYB5a, and VvMYB5b (from *Vitis*), as well as the *Passiflora* R2R3-MYB sequence. PhPH4 is expressed in the petal epidermis and activates vacuolar acidification in petunia [50]. VvMYB5a and VvMYB5b genes are involved in the regulation of anthocyanin biosynthesis during grape berry development [51]. 

WD40 proteins are highly conserved and can be found in organisms that do not biosynthesize anthocyanins as algae, fungi, and animals [78, 79]. In plants, these proteins are involved in a plethora of developmental and biochemical functions. As an example, the *Arabidopsis* TRANSPARENT TESTA GLABRA 1 (TTG1), which is a WD40 protein, is involved in regulating trichome formation, anthocyanin biosynthesis, seed coat pigmentation, and seed coat mucilage production. A common feature of WD40 repeat proteins is that they facilitate protein-protein interactions between the MYB and bHLH proteins [22, 79].

The alignment of the *Passiflora* WD40 protein sequence with other known WD40s from different plant species revealed the presence of conserved WD40 motifs in the C-terminal region (Figure 9). The phylogenetic tree constructed based on this alignment is shown in Figure 10. The results indicated that the monocot sequences ZmPAC1 and OsWD clustered together, whereas the eudicot WD40s known to function as anthocyanin regulators were grouped into a different clade, with *Passiflora *WD40 being closely related to the *Ricinus communis* protein (RcWD, Table 1 and Figure 10). Although WD40 proteins are required to regulate anthocyanins and proanthocyanidin together with MYB and bHLH transcription factors, their potential involvement in other biological processes is enormous, therefore, it is premature to say what functions PACEPE3007G07.g might perform in *Passiflora*.

The fact that no putative homologs to bHLH transcription factors were found in the PASSIOMA database may reflect the high degree of novelty of most of the libraries of the PASSIOMA project indicating that full gene expression spectra was not completely achieved [27]. Perhaps a more deep sequencing effort would reveal that such homologs are indeed expressed in *Passiflora* flowers, as these elements are generally essential to MYB-WD40 protein complex stability [30–32].

## 5. Conclusions and Perspectives

We took the first steps toward the understanding of the molecular processes involved in the biosynthesis of anthocyanins in* Passiflora* that could account for the differences in pollinator preferences found in the genus. We identified 15 putative coding sequences derived from two distinct *Passiflora* species (*P. edulis* and *P. suberosa*) expressed in developing flower buds and potentially involved in the anthocyanin biosynthetic pathway. Comparisons of deduced amino acid sequences from the 15 *Passiflora* cDNAs with selected sequences from other plant species revealed strong similarity with genes that encode key elements involved in the biosynthesis (8 sequences), transcriptional regulation (2 sequences), and transport (5 sequences) of anthocyanin molecules.

Needed research concerning the determination of temporal and spatial expression patterns of all these *Passiflora *putative anthocyanin-related genes presented here are already ongoing in our group. We expect that future work on the manipulation of their expression patterns, using transgenic approaches, will help us to unravel important aspects relating anthocyanin biosynthesis, flower pigmentation, and flower pollination in rapidly changing tropical environments.

## Supplementary Material

The Supplementary material consists of Tables S1 to S6 that contain the GenBank accession numbers of proteins used in the Neigbour-joining phylogenetic trees presented in Figures 2, 4–6, 8 and 10.

## Figures and Tables

**Table 1:** Putative *Passiflora* homologs of genes encoding elements of the anthocyanin biosynthetic pathway.

Enzyme	* Passiflora* AS*	First BLAST hit	*e*-value	ID/SM
CHS	PACEPE3010G11.g	ABD24222 *CHS Populus alba *	7*e* ^−72^	85/90
	PACEPE3014B06.g	ABC86919 CHS *Populus alba *	9*e* ^−67^	77/84
	PACEPE3007G06.g	XP_002305446 CHS-like *Populus trichocarpa *	7*e* ^−121^	84/92
	PACEPE3023H10.g	XP_002326830 CHS-like *Populus trichocarpa *	3*e* ^−110^	82/91
	PACEPS7017D03.g	AAQ62589 CHS3 *Glycine Max *	1*e* ^−98^	82/88
DFR	PACEPE3003G04.g	XP_002307667 DFR2 *Populus trichocarpa *	1*e* ^−95^	82/94
GT	PACEPE3030G03.g	XP_002532899 UFGT *Ricinus communis *	6*e* ^−31^	53/70
	PACEPS7021H07.g	XP_002518725 UFGT *Ricinus communis *	8*e* ^−57^	56/72
GST	PACEPE3013H01.g	AF048978 GST *Glycine Max *	7*e* ^−52^	79/90
	PACEPE3007A05.g	XM_002519342 GST theta *Ricinus communis *	4*e* ^−52^	77/89
	PACEPE3018F08.g	ADB11335 GSTF7 phi *Populus trichocarpa *	2*e* ^−82^	68/83
	PACEPS4006H06.g	ADB11332 GSTF4 phi *Populus trichocarpa *	2*e* ^−61^	63/78
	PACEPS7023B03.g	AF243378 GST 23 *Glycine max *	1*e* ^−51^	80/88
MYB	PACEPS7022E07.g	XP_002530824 R2R3 MYB *Ricinus communis *	7*e* ^−80^	88/91
WD40	PACEPE3007G07.g	XP_002512788 WD-repeat protein *Ricinus communis *	3*e* ^−124^	92/96

Abbreviations: CHS: chalcone synthase; DFR: dihydroflavonol 4-reductase; GT: glucosyltransferase and GST: glutathione S-transferase.

Using the BLASTp algorithm [29].

*AS: assembled sequence. Codes refer to the longest cDNA clone. PACEPE: *Passiflora edulis*; PACEPS: *Passiflora suberosa*.

ID/SM: identity/similarity (both based on the amino acid sequence) with the first BLAST hit.

## References

[1] Harborne JB (1994). *The Flavonoids: Advances in Research Since 1986*.

[2] Grotewold E (2006). The genetics and biochemistry of floral pigments. *Annual Review of Plant Biology*.

[3] Rausher MD (2008). Evolutionary transitions in floral color. *International Journal of Plant Sciences*.

[4] Quattrocchio F, Wing J, van der Woude K (1999). Molecular analysis of the *anthocyanin*2 gene of Petunia and its role in the evolution of flower color. *Plant Cell*.

[5] Spelt C, Quattrocchio F, Mol JNM, Koes R (2000). *Anthocyanin*1 of Petunia encodes a basic helix-loop-helix protein that directly activates transcription of structural anthocyanin genes. *Plant Cell*.

[6] Heller W, Forkmann G, Britsch L, Grisebach H (1985). Enzymatic reduction of (+)-dihydroflavonols to flavan-3,4-cis-diols with flower extracts from *Matthiola incana* and its role in anthocyanin biosynthesis. *Planta*.

[7] Stich K, Eidenberger T, Wurst F, Forkmann G (1992). Enzymatic conversion of dihydroflavonols to flavan-3,4-diols using flower extracts of *Dianthus caryophyllus* L. (carnation). *Planta*.

[8] Davies KM, Bradley JM, Schwinn KE, Markham KR, Podivinsky E (1993). Flavonoid biosynthesis in flower petals of five lines of lisianthus (*Eustoma grandiflorum* Grise.). *Plant Science*.

[9] Helariutta Y, Elomaa P, Kotilainen M, Seppänen P, Teeri TH (1993). Cloning of cDNA coding for dihydroflavonol-4-reductase (DFR) and characterization of dfr expression in the corollas of *Gerbera hybrida* var. Regina (Compositae). *Plant Molecular Biology*.

[10] Grotewold E, Drummond BJ, Bowen B, Peterson T (1994). The myb-homologous P gene controls phlobaphene pigmentation in maize floral organs by directly activating a flavonoid biosynthetic gene subset. *Cell*.

[11] Hernandez JM, Heine GF, Irani NG (2004). Different mechanisms participate in the R-dependent activity of the R2R3 MYB transcription factor C1. *The Journal of Biological Chemistry*.

[12] Schwinn K, Venail J, Shang Y (2006). A small family of MYB-regulatory genes controls floral pigmentation intensity and patterning in the Genus *antirrhinum*. *Plant Cell*.

[13] Morita Y, Saitoh M, Hoshino A, Nitasaka E, Iida S (2006). Isolation of cDNAs for R2R3-MYB, bHLH and WDR transcriptional regulators and identification of c and ca mutations conferring white flowers in the Japanese morning glory. *Plant and Cell Physiology*.

[14] Park KI, Ishikawa N, Morita Y, Choi JD, Hoshino A, Iida S (2007). A bHLH regulatory gene in the common morning glory, Ipomoea *purpurea*, controls anthocyanin biosynthesis in flowers, proanthocyanidin and phytomelanin pigmentation in seeds, and seed trichome formation. *Plant Journal*.

[15] Holton TA, Cornish EC (1995). Genetics and biochemistry of anthocyanin biosynthesis. *Plant Cell*.

[16] Koes R, Verweij W, Quattrocchio F (2005). Flavonoids: a colorful model for the regulation and evolution of biochemical pathways. *Trends in Plant Science*.

[17] Davies K (2004). Plant pigments and their manipulation. *Annual Plant Reviews*.

[18] Lee HS, Hong V (1992). Chromatographic analysis of anthocyanins. *Journal of Chromatography*.

[19] Harborne JB, Mabry TJ, Mabry H (1975). *The Flavonoids*.

[20] Mol J, Grofewold E, Koes R (1998). How genes paint flowers and seeds. *Trends in Plant Science*.

[21] Winkel-Shirley B (2001). Flavonoid biosynthesis. A colorful model for genetics, biochemistry, cell biology, and biotechnology. *Plant Physiology*.

[22] Ramsay NA, Glover BJ (2005). MYB-bHLH-WD40 protein complex and the evolution of cellular diversity. *Trends in Plant Science*.

[23] Lepiniec L, Debeaujon I, Routaboul JM (2006). Genetics and biochemistry of seed flavonoids. *Annual Review of Plant Biology*.

[24] Ulmer T, MacDougal JM (2004). *Passiflora, Passion Flowers of the World*.

[25] Halim MM, Collins RP (1970). Anthocyanins of *Passiflora quadrangularis*. *Bulletin of the Torrey Botanical Club*.

[26] KidØy L, Nygård AM, Andersen ØM, Pedersen AT, Aksnes DW, Kiremire BT (1997). Anthocyanins in fruits of *Passiflora edulis* and *P. suberosa*. *Journal of Food Composition and Analysis*.

[27] Dornelas MC, Tsai SM, Rodriguez APM, Teixeira da Silva JA (2006). Expressed sequence tags of genes involved in the flowering process of *Passiflora* spp.. *Floriculture, Ornamental and Plant Biotechnology*.

[28] Varassin IG, Trigo JR, Sazima M (2001). The role of nectar production, flower pigments and odour in the pollination of four species of *Passiflora* (Passifloraceae) in south-eastern Brazil. *Botanical Journal of the Linnean Society*.

[29] Altschul SF, Gish W, Miller W, Myers EW, Lipman DJ (1990). Basic local alignment search tool. *Journal of Molecular Biology*.

[30] Nakatsuka T, Abe Y, Kakizaki Y, Yamamura S, Nishihara M (2007). Production of red-flowered plants by genetic engineering of multiple flavonoid biosynthetic genes. *Plant Cell Reports*.

[31] Hanumappa M, Choi G, Ryu S, Choi G (2007). Modulation of flower colour by rationally designed dominant-negative chalcone synthase. *Journal of Experimental Botany*.

[32] Martens S, Preuß A, Matern U (2010). Multifunctional flavonoid dioxygenases: flavonol and anthocyanin biosynthesis in *Arabidopsis thaliana* L. *Phytochemistry*.

[33] Huang X, Madan A (1999). CAP3: a DNA sequence assembly program. *Genome Research*.

[34] Hall TA (1999). BioEdit: a user-friendly biological sequence alignment editor and analysis program for Windows 95/98/NT. *Nucleic Acids Symposium Series*.

[35] Hunter S, Apweiler R, Attwood TK (2009). InterPro: the integrative protein signature database. *Nucleic Acids Research*.

[36] Thompson JD, Gibson TJ, Plewniak F, Jeanmougin F, Higgins DG (1997). The CLUSTAL X windows interface: flexible strategies for multiple sequence alignment aided by quality analysis tools. *Nucleic Acids Research*.

[37] Kumar S, Tamura K, Nei M (2004). MEGA3: integrated software for molecular evolutionary genetics analysis and sequence alignment. *Briefings in Bioinformatics*.

[38] Saitou N, Nei M (1987). The neighbor-joining method: a new method for reconstructing phylogenetic trees. *Molecular Biology and Evolution*.

[39] Moore MJ, Soltis PS, Bell CD, Burleigh JG, Soltis DE (2010). Phylogenetic analysis of 83 plastid genes further resolves the early diversification of eudicots. *Proceedings of the National Academy of Sciences of the United States of America*.

[40] Ageez A, Kazama Y, Sugiyama R, Kawano S (2005). Male-fertility genes expressed in male flower buds of *Silene latifolia* include homologs of anther-specific genes. *Genes and Genetic Systems*.

[41] Jiang C, Schommer CK, Kim SY, Suh DY (2006). Cloning and characterization of chalcone synthase from the moss, *Physcomitrella patens*. *Phytochemistry*.

[42] Beld M, Martin C, Huits H, Stuitje AR, Gerats AGM (1989). Flavonoid synthesis in *Petunia hybrida*: partial characterization of dihydroflavonol-4-reductase genes. *Plant Molecular Biology*.

[43] Johnson ET, Ryu S, Yi H, Shin B, Cheong H, Choi G (2001). Alteration of a single amino acid changes the substrate specificity of dihydroflavonol 4-reductase. *Plant Journal*.

[44] Shimada N, Sasaki R, Sato S (2005). A comprehensive analysis of six dihydroflavonol 4-reductases encoded by a gene cluster of the *Lotus japonicus* genome. *Journal of Experimental Botany*.

[45] Kovinich N, Saleem A, Arnason JT, Miki B (2010). Functional characterization of a UDP-glucose:flavonoid 3-O- glucosyltransferase from the seed coat of black soybean (*Glycine max* (L.) Merr.). *Phytochemistry*.

[46] Dixon DP, Lapthorn A, Edwards R (2002). Plant glutathione transferases. *Genome Biology*.

[47] Borovsky Y, Oren-Shamir M, Ovadia R, De Jong W, Paran I (2004). The A locus that controls anthocyanin accumulation in pepper encodes a MYB transcription factor homologous to Anthocyanin2 of Petunia. *Theoretical and Applied Genetics*.

[48] Zimmermann IM, Heim MA, Weisshaar B, Uhrig JF (2004). Comprehensive identification of *Arabidopsis thaliana* MYB transcription factors interacting with R/B-like BHLH proteins. *Plant Journal*.

[49] Grotewold E, Sainz MB, Tagliani L, Hernandez JM, Bowen B, Chandler VL (2000). Identification of the residues in the Myb domain of maize C1 that specify the interaction with the bHLH cofactor R. *Proceedings of the National Academy of Sciences of the United States of America*.

[50] Quattrocchio F, Verweij W, Kroon A, Spelt C, Mol J, Koes R (2006). PH4 of petunia is an R2R3 MYB protein that activates vacuolar acidification through interactions with basic-helix-loop-helix transcription factors of the anthocyanin pathway. *Plant Cell*.

[51] Deluc L, Bogs J, Walker AR (2008). The transcription factor VvMYB5b contributes to the regulation of anthocyanin and proanthocyanidin biosynthesis in developing grape berries. *Plant Physiology*.

[52] Funa N, Ozawa H, Hirata A, Horinouchi S (2006). Phenolic lipid synthesis by type III polyketide synthases is essential for cyst formation in *Azotobacter vinelandii*. *Proceedings of the National Academy of Sciences of the United States of America*.

[53] Funa N, Awakawa T, Horinouchi S (2007). Pentaketide resorcylic acid synthesis by type III polyketide synthase from *Neurospora crassa*. *The Journal of Biological Chemistry*.

[54] Durbin ML, McCaig B, Clegg MT (2000). Molecular evolution of the chalcone synthase multigene family in the morning glory genome. *Plant Molecular Biology*.

[55] Koduri PKH, Gordon GS, Barker EI, Colpitts CC, Ashton NW, Suh DY (2010). Genome-wide analysis of the chalcone synthase superfamily genes of *Physcomitrella patens*. *Plant Molecular Biology*.

[56] Wu S, O’Leary SJB, Gleddie S, Eudes F, Laroche A, Robert LS (2008). A chalcone synthase-like gene is highly expressed in the tapetum of both wheat (*Triticum aestivum* L.) and triticale (x Triticosecale Wittmack). *Plant Cell Reports*.

[57] Koes RE, Spelt CE, van den Elzen PJM, Mol JNM (1989). Cloning and molecular characterization of the chalcone synthase multigene family of *Petunia hybrida*. *Gene*.

[58] Jiang C, Kim SY, Suh DY (2008). Divergent evolution of the thiolase superfamily and chalcone synthase family. *Molecular Phylogenetics and Evolution*.

[59] Forkmann G, Ruhnau B (1987). Distinct substrate specificity of dihydroflavonol-4-reductase from flowers of *Petunia hybrida*. *Zeitschrift für Naturforschung—Section C: Biosciences*.

[60] Rudd S (2003). Expressed sequence tags: alternative or complement to whole genome sequences?. *Trends in Plant Science*.

[61] Ogata J, Itoh Y, Ishida M, Yoshida H, Ozeki Y (2004). Cloning and heterologous expression of cDNAs encoding flavonoid glucosyltransferases from Dianthus caryophyllus. *Plant Biotechnology*.

[62] Isayenkova J, Wray V, Nimtz M, Strack D, Vogt T (2006). Cloning and functional characterisation of two regioselective flavonoid glucosyltransferases from *Beta vulgaris*. *Phytochemistry*.

[63] Alfenito MR, Souer E, Goodman CD (1998). Functional complementation of anthocyanin sequestration in the vacuole by widely divergent glutathione S-transferases. *Plant Cell*.

[64] Marrs KA, Alfenito MR, Lloyd AM, Walbot V (1995). A glutathione S-transferase involved in vacuolar transfer encoded by the maize gene Bronze-2. *Nature*.

[65] Kitamura S, Shikazono N, Tanaka A (2004). TRANSPARENT TESTA 19 is involved in the accumulation of both anthocyanins and proanthocyanidins in *Arabidopsis*. *Plant Journal*.

[66] Loyall L, Uchida K, Braun S, Furuya M, Frohnmeyer H (2000). Glutathione and a UV light-induced glutathione S-transferase are involved in signaling to chalcone synthase in cell cultures. *Plant Cell*.

[67] Meyer RC, Goldsbrough PB, Woodson WR (1991). An ethylene-responsive flower senescence-related gene from carnation encodes a protein homologous to glutathione s-transferases. *Plant Molecular Biology*.

[68] Itzhaki H, Maxson JM, Woodson WR (1994). An ethylene-responsive enhancer element is involved in the senescence- related expression of the carnation glutathione-S-transferase (GST1) gene. *Proceedings of the National Academy of Sciences of the United States of America*.

[69] Elomaa P, Uimari A, Mehto M, Albert VA, Laitinen RAE, Teeri TH (2003). Activation of anthocyanin biosynthesis in *Gerbera hybrida* (Asteraceae) suggests conserved protein-protein and protein-promoter interactions between the anciently diverged monocots and eudicots. *Plant Physiology*.

[70] Mathews H, Clendennen SK, Caldwell CG (2003). Activation tagging in tomato identifies a transcriptional regulator of anthocyanin biosynthesis, modification, and transport. *Plant Cell*.

[71] Stracke R, Werber M, Weisshaar B (2001). The R2R3-MYB gene family in *Arabidopsis thaliana*. *Current Opinion in Plant Biology*.

[72] Lin-Wang K, Bolitho K, Grafton K (2010). An R2R3 MYB transcription factor associated with regulation of the anthocyanin biosynthetic pathway in Rosaceae. *BMC Plant Biology*.

[73] Yamagishi M, Shimoyamada Y, Nakatsuka T, Masuda K (2010). Two R2R3-MYB genes, homologs of petunia AN2, regulate anthocyanin biosyntheses in flower tepals, tepal spots and leaves of asiatic hybrid Lily. *Plant and Cell Physiology*.

[74] Jiang C, Gu X, Peterson T (2004). Identification of conserved gene structures and carboxy-terminal motifs in the Myb gene family of *Arabidopsis* and *Oryza sativa* L. ssp. indica. *Genome Biology*.

[75] Kobayashi S, Ishimaru M, Hiraoka K, Honda C (2002). Myb-related genes of the Kyoho grape (Vitis labruscana) regulate anthocyanin biosynthesis. *Planta*.

[76] Walker AR, Lee E, Bogs J, McDavid DAJ, Thomas MR, Robinson SP (2007). White grapes arose through the mutation of two similar and adjacent regulatory genes. *Plant Journal*.

[77] Gonzalez A, Zhao M, Leavitt JM, Lloyd AM (2008). Regulation of the anthocyanin biosynthetic pathway by the TTG1/bHLH/Myb transcriptional complex in *Arabidopsis* seedlings. *Plant Journal*.

[78] de Vetten N, Quattrocchio F, Mol J, Koes R (1997). The an11 locus controlling flower pigmentation in petunia encodes a novel WD-repeat protein conserved in yeast, plants, and animals. *Genes and Development*.

[79] Brueggemann J, Weisshaar B, Sagasser M (2010). A WD40-repeat gene from Malus × domestica is a functional homologue of *Arabidopsis thaliana* TRANSPARENT TESTA GLABRA1. *Plant Cell Reports*.

