# Supplementary material for: A Genomic Approach to Study Anthocyanin Synthesis and Flower Pigmentation in Passionflowers
Source: J Nucleic Acids. 2011 May 5;2011:371517. doi: 10.4061/2011/371517 (PMC3137904; doi:10.4061/2011/371517)
Supplement: Supplementary file 1 — The Supplementary material consists of Tables S1 to S6 that contain the GenBank accession numbers of proteins used in the Neigbour-joining phylogenetic trees presented in Figures 2, 4–6, 8 and 10. [file 371517.f1.doc]

TABLE S1. GenBank acession numbers of proteins used in Figure 2.

CHALCONE SYNTASE (CHS)

| Species | Protein | GenBank acession |
| --- | --- | --- |
|  |  |  |
| *Synechococcus* sp. | SyPKS | NP_897086 |
| *Pinus radiata* | PrCHS | AAB80804 |
| *Oryza sativa* | OsCHSL | AAL59036 |
| *Hypericum perforatum* | HpCHSL | ABP98922 |
| *Nicotiana benthamiana* | NbCHS | ABN80439 |
| *Nicotiana tabacum* | NtCHS | AAK49457 |
| *Nicotiana sylvestris* | NsCHSL | CAA74847 |
| *Hordeum vulgare* | HvCHSL | AAV49989 |
| *Triticum aestivum* | TaCHSL | CAJ15412 |
| *Aegilops tauschii* | AtCHSL | CAJ13966 |
| *Arabidopsis halleri* | AhCHSL | AAZ23686 |
| *Arabidopsis thaliana* | AthCHS | CAI30411 |
| *Arabidopsis thaliana* | AthCHSL1 | NM_100085 |
| *Arabidopsis thaliana* | AthCHSL2 | NM_119651 |
| *Arabidopsis thaliana* | AthCHSL3 | AAM63363 |
| *Vitis vinifera* | VvCHSL | CAO47307 |
| *Silene latifolia* | SlCHSL | BAE80096 |
| *Equisetum arvense* | EaCHS | AB030004 |
| *Psilotum nudum* | PnCHS | AB022682 |
| *Populus trichocarpa* | PtCHS1 | ABK95168 |
| *Populus trichocarpa* | PtCHL1 | XP_00230548 |
| *Populus trichocarpa* | PtCHL2 | XP_002326830 |
| *Medicago ativa* | MsCHS | P30074 |
| *Pinus sylvestris* | PsCHS | X60754 |
| *Rheum palmatum* | RpCHS | DQ205352 |
| *Zea mays* | ZmCHS | CAA42763 |
| *Oryza sativa* | OsCHS2 | BAA19186 |
| *Oryza sativa* | OsCHS | BAB39764 |
| *Physcomitrella patens* | PpCHS01 | XP_001756277 |
| *Physcomitrella patens* | PpCHS10 | ABU87504 |
| *Physcomitrella patens* | PpCHS11 | XP_001781520 |
| *Petunia hybrida* | PhCSHa | P08894 |
| *Petunia hybrida* | PHCSHj | P22928 |

TABLE S2. GenBank acession numbers of proteins used in Figure 4.

DIHYDROFLAVONOL4-O REDUCTASE (DFR)

| | Species | Protein | GenBank acession | | --- | --- | --- | |  |  |  | | *Antirrhinum majus* | AmDFR | X15536 | | *Arabidopsis thaliana* | AtDFR | AB033294 | | *Bromheadia finlaysoniana* | BfDFR | AF007096 | | *Callistephus chinensis* | CcDFR | Z67981 | | *Camellia sinensis* | CsDFR | AB018686 | | *Cymbidium hybrid* | ChDFR | AF017451 | | *Daucus carota* | DcDFR | AF18427 | | *Dianthus caryophyllus* | DicDFR | Z67983 | | *Forsythia x intermedia* | FiDFR | Y09127 | | *Fragaria x ananassa* | FaDFR | AF029685 | | *Gentiana triflora* | GtDFR | D85185 | | *Gerbera hybrida* | GhDFR | Z17221 | | *Hordeum vulgare* | HvDFR | S69616 | | *Ipomoea nil* | InDFRA | AB006793 | | *Ipomoea purpurea* | IpDFRA | AB011667 | | *Lilium_hybrid* | LhDFR | AF169801 | | *Lotus corniculatus* | LcDFR3 | AF117263 | | *Lotus japonicus* | LjDFR1 | AB162109 | | *Lotus japonicus* | LjDFR2 | AB162110 | | *Lotus japonicus* | LjDFR3 | AB162111 | | *Lotus japonicus* | LjDFR4a | AB162112 | | *Lotus japonicus* | LjDFR5 | AB162114 | | *Solanum lycopersicon* | SlDFR | Z18277 | | *Malus domestica* | MdDFR | AF117268 | | *Medicago truncatula DFR1* | MtDFR1 | AY38934 | | *Medicago truncatula DFR2* | MtDFR2 | AY389347 | | *Oryza sativa* | OsDFR | AB003496 | | *Perilla frutescens* | PfDFR | AB002817 | | *Petunia x hybrida* | PhDFR | AF233639 | | *Populus trichocarpa DFR1* | PtDFR1 | XM_00230072 | | *Populus trichocarpa DFR2* | PtDFR2 | XP_00230766 | | *Rosa hybrida* | RhDFR | D85102 | | *Solanum tuberosum* | StDFR | AF449422 | | *Torenia hybrida* | ThDFR | AB012924 | | *Triticum aestivum* | TaDFR | AAQ77347 | | *Triticum monococcum* | TmDFR | AF434703 | | *Vaccinium macrocarpon* | VmDFR1 | AF483835 | | *Vaccinium macrocarpon* | VmDFR2 | AF483836 | | *Vitis vinifera* | VvDFR | Y11749 | |  |  |
| --- | --- | --- | --- | --- | --- | --- | --- | --- | --- | --- | --- | --- | --- | --- | --- | --- | --- | --- | --- | --- | --- | --- | --- | --- | --- | --- | --- | --- | --- | --- | --- | --- | --- | --- | --- | --- | --- | --- | --- | --- | --- | --- | --- | --- | --- | --- | --- | --- | --- | --- | --- | --- | --- | --- | --- | --- | --- | --- | --- | --- | --- | --- | --- | --- | --- | --- | --- | --- | --- | --- | --- | --- | --- | --- | --- | --- | --- | --- | --- | --- | --- | --- | --- | --- | --- | --- | --- | --- | --- | --- | --- | --- | --- | --- | --- | --- | --- | --- | --- | --- | --- | --- | --- | --- | --- | --- | --- | --- | --- | --- | --- | --- | --- | --- | --- | --- | --- | --- | --- | --- | --- | --- | --- | --- | --- |

TABLE S3. GenBank acession numbers of proteins used in Figure 5.

GLUCOSYLTRANSFERASE (GT)

| | Species | Protein | GenBank acession | | --- | --- | --- | |  |  |  | | *Glycine Max* | UGT78K1 | GU434274 | | *Glycine Max* | GmIF7GT | AB292164 | | *Clitoria ternatea* | Ct3GT-A | AB185904 | | *Arabidopsis thaliana* | UGT78D2 | NM_121711 | | *Verbena x hybrida* | HGT8 | AB013598 | | *Perilla frutescens* | PfUA5GT | AB013596 | | *Gentiana triflora* | GtUF3GT | D85186 | | *Gentiana triflora* | Gt5GT7 | AB363839 | | *Vitis vinifera* | VvGT1 | AF00037 | | *Iris hollandica* | Ih3GT | AB161175 | | *Dianthus caryophyllus* | DicGT1 | AB191245 | | *Dianthus caryophyllus* | DicGT2 | AB191246 | | *Dianthus caryophyllus* | DicGT3 | AB191247 | | *Dianthus caryophyllus* | DicGT4 | AB191248 | | *Dianthus caryophyllus* | DicGT5 | AB191249 | | *Rosa hybrid* | RhGT1 | AB201048 | | *Rosa hybrid* | RhGT2 | AB201049 | | *Rosa hybrid* | RhGT3 | AB201050 | | *Solanum sogarandinum* | Ssci17 | AY033489 | | *Fragaria x ananassa* | FaGT1 | AY663784 | | *Fragaria x ananassa* | FaGT6 | DQ289587 | | *Fragaria x ananassa* | FaGT7 | DQ289588 | | *Fragaria x ananassa* | FaUF3GT | AY695815 | | *Medicago truncatula* | UGT73C8 | DQ875459 | | *Medicago truncatula* | UGT88E1 | DQ875460 | | *Medicago truncatula* | UGT88E2 | DQ875461 | | *Medicago truncatula* | UGT71G1 | AAW56092 | | *Medicago truncatula* | UGT78G1 | DQ875464 | | *Citrus x paradisi* | Cp1UF3GT | GQ141630 | | *Citrus x paradisi* | Cp2UF3GT | GQ141631 | | *Forsythia x intermedia* | FiUF3GT | AF127218 | | *Malus x domestica* | MdUF3GT | DQ156906 | | *Perilla frutescens* | PfUF3GT | AB002818 | | *Solanum tuberosum* | StUF3GT | AY954034 | | *Scutellaria baicalensis* | SbUF7GT | AB031274 | | *Beta vulgaris* | BvFGT | AY526080 | | *Beta vulgaris* | BvFGT2 | AY526081 | | *Arabidopsis thaliana* | UGT73C6 | NM_129234 | | *Oryza sativa* | UGT707A3 | BAC83989 | | *Oryza sativa* | UGT709A4 | BAC80066 | | *Crocus sativus* | CsGT45 | FJ194947 | | *Populus trichocarpa* | PtFGT1 | EEE95837 | | *Populus trichocarpa* | PtFGT2 | EEE74119 | |  |  |
| --- | --- | --- | --- | --- | --- | --- | --- | --- | --- | --- | --- | --- | --- | --- | --- | --- | --- | --- | --- | --- | --- | --- | --- | --- | --- | --- | --- | --- | --- | --- | --- | --- | --- | --- | --- | --- | --- | --- | --- | --- | --- | --- | --- | --- | --- | --- | --- | --- | --- | --- | --- | --- | --- | --- | --- | --- | --- | --- | --- | --- | --- | --- | --- | --- | --- | --- | --- | --- | --- | --- | --- | --- | --- | --- | --- | --- | --- | --- | --- | --- | --- | --- | --- | --- | --- | --- | --- | --- | --- | --- | --- | --- | --- | --- | --- | --- | --- | --- | --- | --- | --- | --- | --- | --- | --- | --- | --- | --- | --- | --- | --- | --- | --- | --- | --- | --- | --- | --- | --- | --- | --- | --- | --- | --- | --- | --- | --- | --- | --- | --- | --- | --- | --- | --- | --- | --- | --- |

TABLE S4. GenBank acession numbers of those proteins used in Figure 6.

GLUTATHIONE S-TRANSFERASE (GST)

| Species | Protein | GenBank acession |
| --- | --- | --- |
|  |  |  |
| *Vitis vinifera* | VvGST1 | AAN85826 |
| *Vitis vinifera* | VvGST2 | EF088687 |
| *Vitis vinifera* | VvGST3 | EF469244 |
| *Vitis vinifera* | VvGST4 | AY971515 |
| *Vitis vinifera* | VvGST5 | EF140721 |
| *Arabidopsis thaliana* | AtPM239 | P42769 |
| *Papaver somniferum* | PsGST1 | AAF22517 |
| *Zea mays* | Bz2 | AAA50245 |
| *Arabidopsis thaliana* | AtGST5 | BAA07917 |
| *Arabidopsis thaliana* | AtPM24 | CAA53051 |
| *Arabidopsis thaliana* | AtERD11 | BAA04553 |
| *Arabidopsis thaliana* | TT19 | NP_197224 |
| *Glycine Max* | GmGST26-A | AAA33973 |
| *Solanum tuberosum* | Stprp-1 | AAA68430 |
| *Nicotiana tabacum* | NtGNT1 | CAA39709 |
| *Nicotiana tabacum* | NtGNT35 | CAA39710 |
| *Nicotiana tabacum* | NtGNT103 | CAA39704 |
| *Nicotiana tabacum* | NtparA | BAA14243 |
| *Nicotiana tabacum* | NtparB | BAA01394 |
| *Nicotiana tabacum* | NtparC | CAA45740 |
| *Nicotiana tabacum* | Nt114 | AAA67894 |
| *Nicotiana tabacum* | Nt107 | CAA39707 |
| *Dianthus caryophyllus* | DcGST1 | AAA72320 |
| *Dianthus caryophyllus* | DcGST2 | AAA5145 |
| *Dianthus caryophyllus* | DcpSR8 | AAA33277 |
| *Silene vulgaris* | SvGST | AAA33930 |
| *Petunia x hybrida* | An9 | CAA68993 |
| *Triticum aestivum* | TaGST1A | CAA39487 |
| *Zea mays* | ZmGSTI | CAA29929 |
| *Zea mays* | ZmGStIII | CAA27957 |
| *Zea mays* | ZmGSTIV | AAA20585 |
| *Populus alba x Populus tremula* | GST18 | BAD91094 |
| *Populus trichocarpa* | PtGST | AAT98377 |
| *Ricinus communis* | RcGST | XP_002509785 |

TABLE S5. GenBank acession numbers of proteins used in Figure 8.

R2R3-MYB

| Species | Protein | GenBank acession |
| --- | --- | --- |
|  |  |  |
| *Vitis vinifera* | VvMYBA1 | AB242302 |
| *Vitis vinifera* | VvMYBA2 | BAD18978 |
| *Vitis vinifera* | VvMYBA3 | BAD18979 |
| *Vitis vinifera* | VvMYB5a | AAS68190 |
| *Vitis vinifera* | VvMYB5b | AAX51291 |
| *Petunia x hybrida* | PhAN2 | AAF66727 |
| *Petunia x hybrida* | PH4 | AAY51377 |
| *Arabidopsis thaliana* | AtPAP1 | AAG42001 |
| *Arabidopsis thaliana* | AtPAP2 | AAG42002 |
| *Arabidopsis thaliana* | MYB113 | NP_176811 |
| *Arabidopsis thaliana* | MYB114 | NP_176812 |
| *Antirrhinum majus* | AmRosea1 | ABB83826 |
| *Antirrhinum majus* | AmRosea2 | ABB83827 |
| *Antirrhinum majus* | AmRosea3 | ABB83828 |
| *Lilium hybrid* | LhMYB6 | BAJ05399 |
| *Lilium hybrid* | LhMYB12 | AB534586 |
| *Zea mays* | ZmC1 | AAA33482 |

TABLE S6. GenBank acession numbers of proteins used in Figure 10.

WD 40

| Species | Protein | GenBank acession |
| --- | --- | --- |
|  |  |  |
| *Petunia x hybrida* | PhAN11 | AAC18914 |
| *Arabidopsis thaliana* | AtTTG1 | CAB45372 |
| *Gossypium hirsutum* | GhTTG1 | AAM95641 |
| *Gossypium hirsutum* | GhTTG3 | AAM95645 |
| *Perilla frutescens* | PfWD | BAB58883 |
| *Zea mays* | ZmPAC1 | AAM76742 |
| *Vitis vinifera* | VvWD | XP_002270777 |
| *Populus trichocarpa* | PtWD | XP_002318500 |
| *Oryza sativa* | OsWD | BAF09665 |
| *Ricinus communis* | RcWD | XP_002512788 |
| *Ipomoea nil* | InWDR1 | BAE94398 |
| *Ipomoea purpurea* | IpWDR1 | BAE94396 |
